# Looks can be Deceiving: A Case Report on the Clinical Value of CA 19-9 in Obstructive Jaundice

**DOI:** 10.7759/cureus.6637

**Published:** 2020-01-12

**Authors:** Fanny Giron, Daniel Alcantar

**Affiliations:** 1 Internal Medicine, MacNeal Hospital, Berwyn, USA

**Keywords:** carbohydrate antigen 19-9, cholangiocarcinoma, benign biliary stricture, cholangitis

## Abstract

Carbohydrate antigen CA19-9 is a marker that has been used for the diagnosis of pancreatic and biliary malignancies, but it can also be present in benign conditions. Herein, we present the case of an 82-year-old female admitted for sepsis secondary to cholangitis, with a CA19-9 level of 12,838.3 U/mL. Initial imaging suggested a potential cholangiocarcinoma, but after multiple studies and biopsies, she was found to have a benign biliary stricture which triggered the cholangitis, explaining the increased CA19-9 levels. Clinicians should keep an open mind when assessing significantly elevated CA19-9 levels.

## Introduction

Carbohydrate antigen 19-9 (CA 19-9) is a tumor marker, a member of the Lewis antigen family, which has been extensively studied as a diagnostic tool for pancreatic and biliary cancers [1].

Whenever present, clinicians must be aware that its elevation may not only be related to malignancies but can also be present in benign conditions associated with jaundice, such as acute cholangitis, choledocholithiasis, benign bile duct strictures, and primary biliary cirrhosis, among others [2].

Herein, we present the case of a patient with an elevated CA 19-9 level, which suggested the presence of a cholangiocarcinoma; however, further work-up revealed the contrary.

## Case presentation

An 82-year-old female with a history of cholecystectomy presented to the emergency department with symptoms of epigastric abdominal pain, fever, and chills. Upon arrival, laboratory data was significant for total bilirubin of 2.6 mg/dL, alkaline phosphatase of 347 U/L, aspartate aminotransferase (AST) of 129 U/L, and alanine aminotransferase (ALT) of 124 U/L. The patient underwent a computed tomography (CT) of the abdomen and pelvis with contrast, which revealed a moderate intrahepatic and extrahepatic ductal dilatation measuring up to 1.5 cm in the common hepatic duct region. Because of these findings, the differential at that time was malignancy causing acute cholangitis. The patient was started on intravenous (IV) antibiotics, and a CA19-9 was found to be significantly elevated at a level of 12,838.3 U/mL.

**Figure 1:**
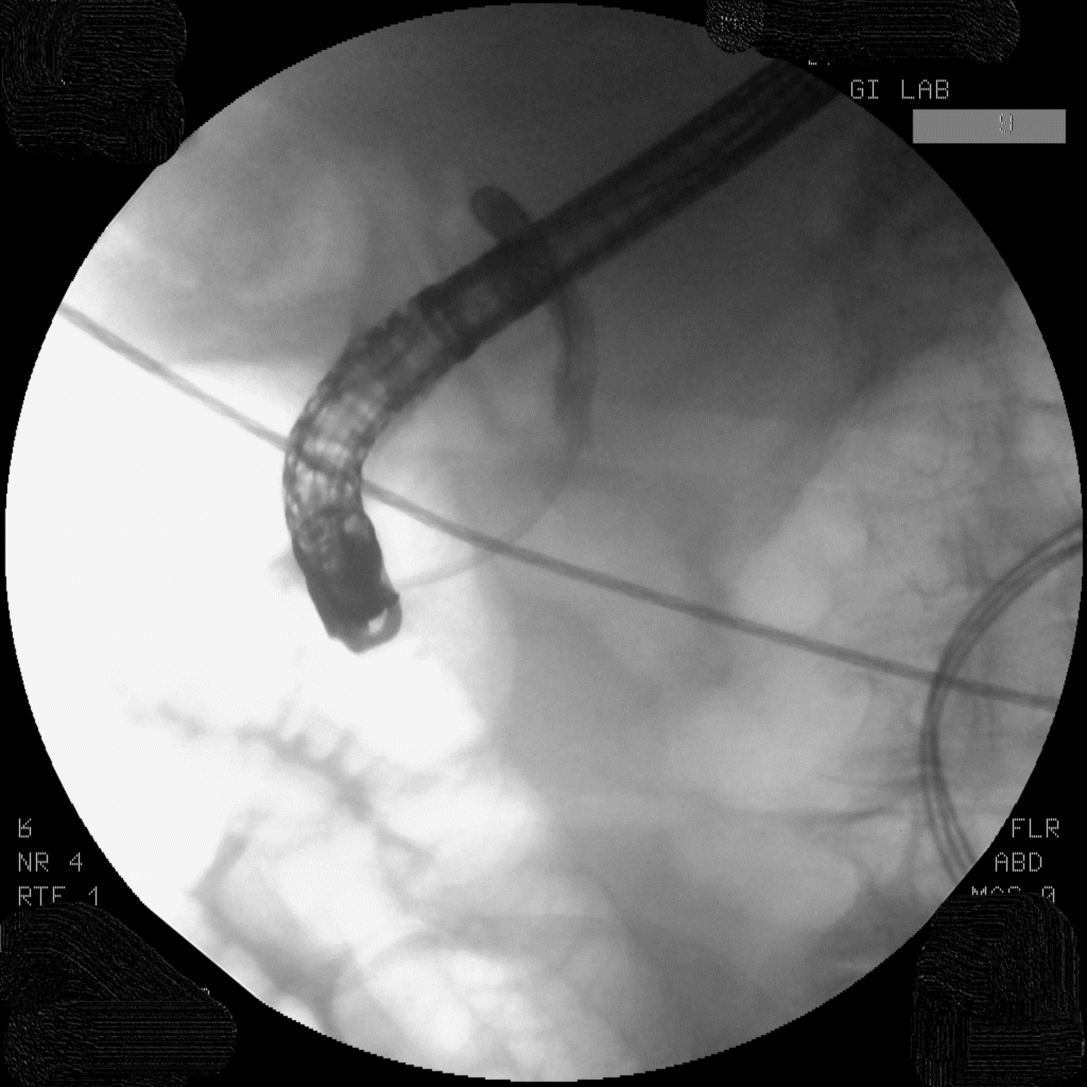
Opacification of the distal CBD without proximal filling noted: Inability to opacify proximal biliary duct CBD, common bile duct

The patient underwent an endoscopic retrograde cholangiopancreatography (ERCP), which revealed the opacification of the distal common bile duct (CBD) without filling of the proximal CBD (Figure 1). 

**Figure 2:**
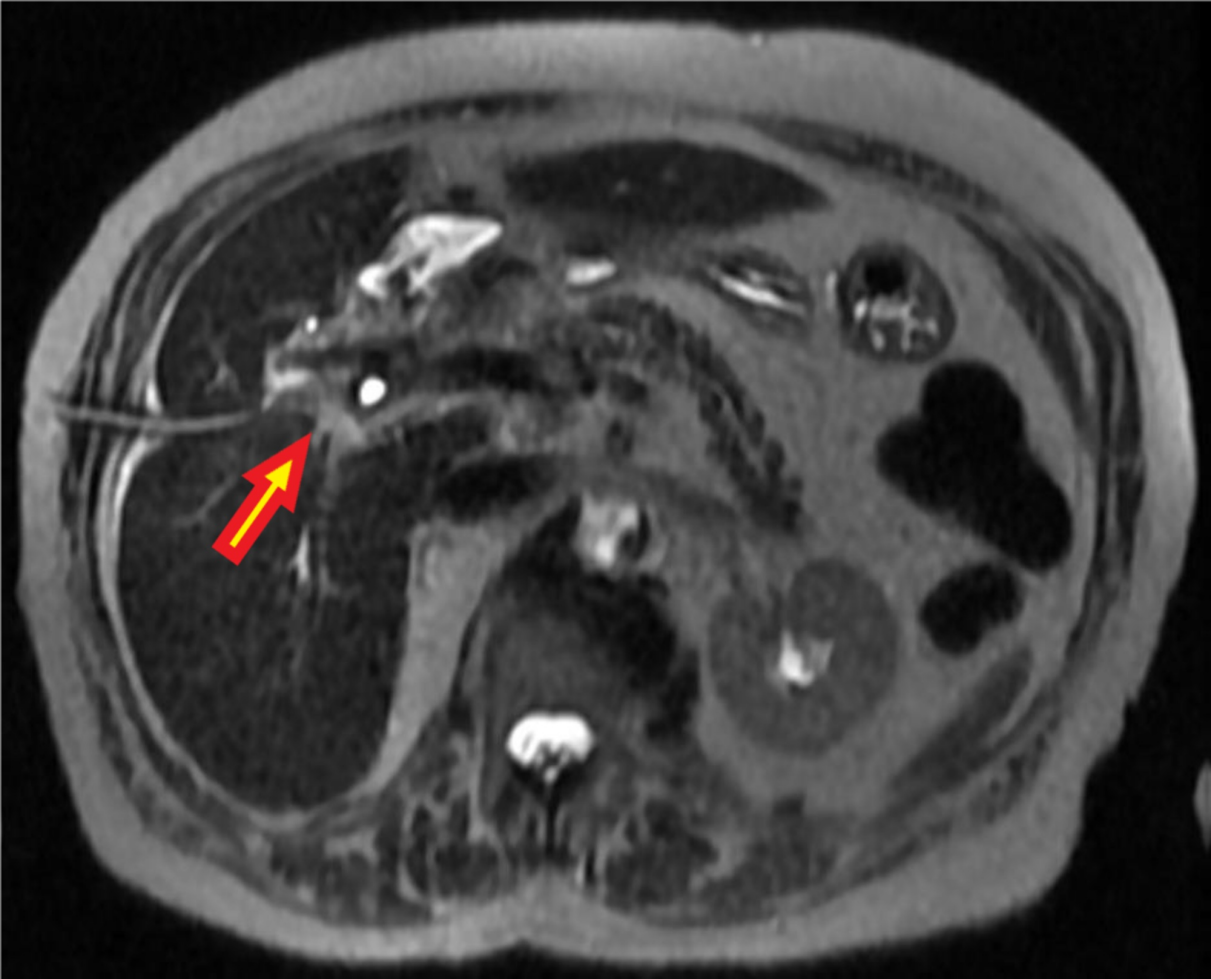
Abrupt cut-off with nonopacification of the distal intrahepatic ducts at the confluence which involves the majority of the length of the common bile duct In this region there is an ill-defined, T2 isointense, markedly enhancing area at the porta hepatis that is suspicious for the underlying mass.

Several attempts to cannulate the proximal CBD were unsuccessful. Because of the filling defect on ERCP, a magnetic resonance cholangiopancreatography (MRCP) was performed, which revealed an abrupt cutoff at the confluence of the right hepatic ducts and no visualization of the common hepatic duct (Figure 2).

A percutaneous transhepatic cholangiography (PTC) was performed, which revealed a mildly prominent intrahepatic biliary tree that was opacified. A brush biopsy of the CBD was obtained, and a biliary drainage catheter was placed. The patient’s pain resolved after PTC drain placement and her CA19-9 level dropped down to 6,012.9 U/ml.

The brush biopsies were inconclusive, but the blood cultures drawn from admission grew E Coli. The patient was treated for cholangitis; however, due to such elevated CA19-9 levels, the concern for cholangiocarcinoma was still high. The patient was transferred to a tertiary center for further evaluation. Records where obtained from the tertiary center and the patient underwent: endoscopic ultrasound (EUS) with a fine needle biopsy from a lymph node (LN) and PTC drain exchange with a bile duct brushing. Again, the biopsy results were inconclusive for malignancy, and the patient was diagnosed with a biliary stricture. Ultimately, a choledocojejunostomy was performed to relieve the biliary stricture. Biopsies obtained during this procedure again revealed no malignancy.

## Discussion

CA 19-9 levels are known to be elevated in malignant conditions such as cholangiocarcinoma and pancreatic, hepatocellular, and ovarian cancer [2]. Normal CA19-9 levels range between 0-37 U/L. This cutoff is currently used to detect pancreatic cancer but has also been studied for cholangiocarcinoma with a sensitivity of 77% to 78% and a specificity of 81% to 84% [3]. When using this marker for cholangiocarcinoma diagnosis, one must consider the effect that obstructive jaundice has over this marker. It has been observed that elevated CA 19 -9 levels can also correlate with other conditions such as strictures and acute cholangitis [4].

Currently, the most common causes of significant elevations of CA 19 -9 in benign biliary pathologies are acute cholangitis and choledocholithiasis, as demonstrated by a recent study by Tsen et al. CA 19-9 levels in acute cholangitis were reported to be as high as 35,500 U/mL and a choledocholithiasis case with a CA19 -9 level of 405,000 K U/L 3. Therefore, CA 19 -9 levels must be interpreted with caution as there is no cut-off value that may help detect malignant versus benign pathologies [3].

Studies have demonstrated some differences between benign and malignant conditions that may help us discern between both. For instance, Mann et al. revealed that CA 19 -9 levels fell in all cases of benign jaundice after biliary drainage, like what was seen in our case. Conversely, CA 19 -9 levels had a diverse response to biliary drainage in malignant conditions [4]. This method should be further studied to determine its validity.

To our knowledge, our case is one of the few that reports significantly elevated CA 19-9 (12,838.3 U/mL) levels in the setting of acute cholangitis due to a benign biliary stricture. Isolated cases of acute cholangitis or benign strictures have been reported to cause elevated CA 19-9 levels, but both etiologies have not been found together in the same patient. 

The mechanism by which cholangitis increases CA 19-9 levels is not entirely elucidated, but it is believed that cholangiocyte irritation increases the inflammatory production of CA 19-9 [3].

## Conclusions

Further studies are needed to determine whether a decrease in CA 19-9 levels after biliary drainage could reliably suggestive of a benign etiology.

Clinicians should be aware of the different etiologies that may increase CA 19 -9 levels; hence, this marker should not be used to dictate the patient’s management, especially considering the advent of new imaging and endoscopic technologies that can accurately separate benign and malignant conditions in the bile duct.
